# High mobility group box 1 release from cholangiocytes in patients with acute-on-chronic liver failure

**DOI:** 10.3892/etm.2014.1904

**Published:** 2014-08-13

**Authors:** HENG XU, HONGXIA LI, YACHAO QU, JUNFU ZHENG, JUN LU

**Affiliations:** 1 Hepatology and Cancer Biotherapy Ward, Beijing You’an Hospital, Capital Medical University, Beijing 100069, P.R. China; 2 Department of Pathophysiology, Anhui Medical University, Hefei, Anhui 230032, P.R. China

**Keywords:** high mobility group box 1, proinflammatory cytokine, acute-on-chronic liver failure, cholangiocytes

## Abstract

High mobility group box chromosomal protein 1 (HMGB1) is an important proinflammatory molecule in a number of inflammatory disorders, but little is known about its role in acute-on-chronic liver failure (ACLF). To elucidate the role of HMGB1 in ACLF, the expression of HMGB1 in liver specimens from patients with ACLF was investigated. Immunohistochemical staining was performed to confirm the expression and subcellular localization of HMGB1 in liver specimens obtained from 13 patients with ACLF caused by hepatitis B virus (HBV) infection, 20 patients with chronic viral hepatitis B and 20 healthy controls. In addition, TFK-1 cells (human cholangiocarcinoma cell line) were stimulated with lipopolysaccharide (LPS) or tumor necrosis factor (TNF)-α. The extracellular level of HMGB1 in the culture medium was then determined by ELISA, and cell viability was also examined. In patients with ACLF caused by HBV infection, HMGB1 was found mainly in the cholangiocytes, and cytoplasmic translocation was observed in the cholangiocytes in the liver specimens. In the TFK-1 cell cultures, HMGB1 levels gradually increased from as early as 4 h after stimulation with LPS or TNF-α until the end of the stimulation. LPS and TNF-α actively induced the cytoplasmic translocation of the HMGB1 protein in TFK-1 cells. These data suggest that HMGB1 plays a critical role in the systemic inflammation associated with ACLF.

## Introduction

Acute-on-chronic liver failure (ACLF) is a recently introduced term that represents the condition of severe acute deterioration due to chronic liver disease. According to Asian Pacific Association for The Study of the Liver (APASL) consensus, ACLF is the acute and rapid deterioration of liver function accompanied by subsequent multiple end-organ failure in a patient with previously well-compensated liver disease due to the effects of a precipitating event (1). In the majority of Asian countries, hepatitis B accounts for 70% of all ACLF cases (1). Although a number of clinical studies have investigated ACLF, the pathophysiology of chronic hepatitis B (CHB)-related ACLF remains poorly understood. Ample evidence suggests that dysregulated inflammation plays an important role due to an imbalanced host response to injury, with a self-perpetuating effect of liver insufficiency (2–6). A previous study suggested that the transition from a stable cirrhotic condition to a sudden, acute decompensation leading to liver failure is caused by an acute systemic inflammatory response that is mainly mediated by cytokines (7). Inflammation of cholangiocytes, which are involved in hyperbilirubinemia and intrahepatic cholestasis, is always observed during ACLF and is considered a marker of poor prognosis.

High mobility group protein (HMG) is a ubiquitous nuclear protein that is expressed in a number of eukaryotic cells. HMG box 1 (HMGB1) belongs to this family, which also includes HMGA, HMGB and HMGN. There are two binding motifs (the A and B boxes) in HMGB1, and the active cytokine domain of HMGB1 is localized to the B box (8,9). HMGB1 is shuttled between the nucleus and cytoplasm via nuclear pores.

As a nuclear protein, HMGB1 stabilizes nucleosomes and enables DNA binding to facilitate gene transcription; however, HMGB1 is also actively released by monocytes and macrophages in response to inflammatory cytokines and is passively released by necrotic or damaged cells (10,11). HMGB1 was recently identified as a late mediator of lethal systemic inflammation diseases, and this role was also demonstrated in an animal model of cytokine-mediated disease (11). Elevated levels of HMGB1 have been observed in patients with acute pancreatitis, acute lung injury, rheumatoid arthritis, hemorrhagic shock, and ischemia-reperfusion injury (12–16), suggesting that HMGB1 is closely associated with these pathogenic processes and may be a central molecule that triggers and maintains the cascading inflammatory reactions.

High levels of HMGB1 have also been detected in the sera of patients with chronic hepatitis (17), acute liver failure (18) and hepatitis B virus-related ACLF (19), demonstrating that HMGB1 release is associated with liver cell damage. In the present study, it was investigated whether HMGB1 is released from cholangiocytes to induce cholangiole inflammation and the exacerbation of intrahepatic cholestasis in ACLF and if such a release can be induced by lipopolysaccharide (LPS) or tumor necrosis factor-α (TNF-α) *in vitro*.

## Materials and methods

### Patients and specimens

Between January 2006 and December 2007, 13 patients with ACLF and 20 patients with CHB were included in this study. ACLF and chronic hepatitis diagnoses fulfilled the criteria of the APASL 2008 consensus (1). Histology was confirmed independently by two pathologists at the You’an Hospital (Beijing, China). All 13 patients underwent liver transplantation. Serological data were collected from archived patient records prior to treatment. Liver function tests included analysis of alanine aminotransferase (ALT), aspartate aminotransferase (AST), total bilirubin (TB), direct bilirubin (DB) and albumin (ALB) levels. The serological marker for hepatitis B was examined prior to ACLF diagnosis, and HBsAg/HBcAg in the liver tissues were routinely detected by immunochemistry in the Department of Pathology. The study conformed to the tenets of the Declaration of Helsinki, and informed written consent was obtained from all patients prior to the study.

### Immunohistochemical staining of liver samples

Liver samples from patients with ACLF and CHB were retrieved from the archives of the Department of Pathology, and normal liver samples were obtained from donors at the liver transplantation center at the You’an Hospital. Serial formalin-fixed, paraffin-embedded samples were deparaffinized in xylene and rehydrated in a series of graded alcohols and distilled water. Endogenous peroxidase activity was quenched by incubation in 3% H_2_O_2_. Prior to immunostaining, the sections were incubated for 12 min in citrate buffer (pH 6.0) in a microwave oven at 99°C to enhance their immunoreactivity. Subsequent to blocking, the sections were incubated at 4°C overnight with rabbit polyclonal anti-HMGB1 antibody (ab-18256; Abcam, Cambridge, MA, USA) or mouse monoclonal anti-CK7 antibody (Zhongshan Jinqiao Biotechnology Co., Beijing, China). Detection was performed using the streptavidin-biotin-peroxidase method with the PV-9000 kit (Zhongshan Jinqiao Biotechnology Co.) according to the manufacturer’s instructions. Diaminobenzidine was employed as a chromogen. Normal liver tissues from adult donors served as negative controls. The slides were counterstained with hematoxylin and mounted. The immunoreactivity of the cholangiocytes was graded based on the percentage of immunopositive cells: +++, >67% of cells stained; ++, 33–67%; +, 5–33%; focal, <5%; and −, no stained cells. Focal staining was also considered negative staining.

### TFK-1 cell culture

The human cholangiocarcinoma cell line TFK-1 (20) was kindly provided by Professor Liu Shun Ai (Ditan Hospital, Beijing, China). TFK-1 cells were maintained in RPMI-1640 medium (HyClone, Logan, UT, USA) supplemented with 100 IU/ml penicillin, 100 mg/ml streptomycin, and 10% fetal bovine serum (Gibco-BRL, Grand Island, NY, USA). The cells were cultured at 37°C in a humid atmosphere of 95% air and 5% CO_2_. The cells were passaged three times per week.

### TFK-1 cell stimulation with LPS and TNF-α

TFK-1 cells were seeded at a density of 5×10^4^ cells/ml in 96-well culture dishes (Corning Inc., Corning, NY, USA) and stimulated with 10, 100 or 500 ng/ml TNF-α (Peprotech, Rocky Hill, NJ, USA) or 1, 10 or 40 μg/ml LPS (Sigma, St. Louis, MO, USA) for 4, 8, 16 or 24 h. Unstimulated cells were used as a control group.

### Cell viability assay

Cell viability was determined using an MTT assay as previously described (21). Briefly, TFK-1 cells were cultured in 96-well plates overnight at a density of 2×10^3^ cells per well. The cells were exposed to LPS or TNF-α at each concentration in triplicate at a final volume of 200 μl. Culture medium was used as a control blank. After 4, 8, 16 or 24 h of culture, the absorbance of each well was measured using a Bio-Rad microplate reader (Bio-Rad, Hercules, CA, USA). The cell viability (%) was calculated according to the following formula: Absorbance of experimental well/absorbance of control well × 100.

### HMGB1 concentration in the culture medium of TFK-1 cells

The concentration of HMGB1 in the medium of the LPS- or TNF-α-treated and control cells was determined using an ELISA assay according to the manufacturer’s instructions (HMGB1 ELISA Kit; R&D systems, Abingdon, UK). The ELISA standards ranged from 78–5,000 pg/ml. All samples were measured in triplicate.

### Cell nuclear-cytoplasmic fractionation and western blot analysis

Cells were harvested, and nuclear-cytoplasmic fractionation was conducted using NE-PER^®^ Nuclear and Cytoplasmic Extraction Reagents (Thermo Fisher Scientific, Waltham, MA, USA) according to the manufacturer’s instructions. Western blot analysis was performed as previously described (22). Equivalent amounts of protein were separated by SDS-PAGE and transferred to polyvinylidene difluoride membranes (Millipore, Bedford, MA, USA). Subsequent to blocking, the membranes were incubated with the appropriate diluted primary antibodies, targeting HMGB1, proliferating cell nuclear antigen (PCNA; Abcam) and β-actin (Sigma). The signal of the target protein was detected using an enhanced chemiluminescence detection system (Pierce Biotechnology, Inc., Rockford, IL, USA) and recorded on film in the linear detection range.

### Statistical analysis

All continuous data are expressed as the mean ± standard deviation. Comparisons between groups were performed using the Student’s t-test or the χ^2^ test, including Fisher’s exact test. Statistical significance was defined as P<0.05. All statistical analyses were performed using SPSS software, version 16.0 for Windows (SPSS, Inc., Chicago, IL, USA).

## Results

### Baseline characteristics of the patients

The baseline characteristics of the 13 patients in the ACLF group and the 20 patients in the CHB group are shown in Table I. The TB (428.76±214.40 μmol/l in ACLF vs. 19.49±13.17 μmol/l in CHB) and DB (232.56±115.99 μmol/l in ACLF vs. 6.89±6.65 μmol/l in CHB) levels were highly elevated and the percentage of prothrombin activity (26.47±11.52% in ACLF vs. 95.06±11.48% in CHB) was extremely low in the ALCF group compared with the CHB group. Of the 13 patients with ACLF, five had encephalopathy (grade II–III), four had ascites, one had gastrointestinal tract hemorrhage and five had infections. These complications are consistent with the diagnosis of ACLF. All patients with CHB were HBsAg/HBcAg positive in liver tissues, and 11 of the 13 patients with ACLF were positive for HBsAg/HBcAg.

### HMGB1 and CK7 expression in patients with ACLF and CHB

**Figure 1:**
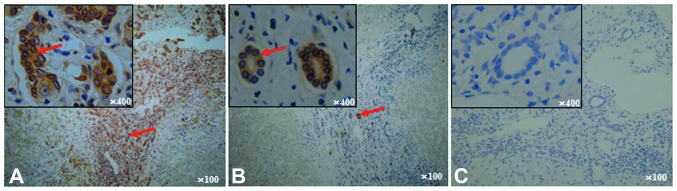
Examples of HMGB1 expression and sub-cellular location in liver specimens. Immunohistochemistry was performed using an anti-HMGB1 antibody visualized with diaminobenzidine (brown). (A) Acute-to-chronic liver failure liver tissue. (B) Chronic hepatitis B liver tissue. (C) Normal liver tissue from a donor. Arrow: cytoplasmic HMGB1 staining in cholangiocytes. Original magnification, ×100 and ×400 (inset). HMGB1, high mobility group box 1.

HMGB1-positive staining was predominantly observed in the cytoplasm and extracellular area of cholangiocytes, particularly in the newly formed cholangiocytes in most inflammatory and portal areas of ACLF. Positive staining for HMGB1 was also observed in the nuclei of certain cholangioles (Fig. 1A), suggesting that nuclear-cytoplasmic HMGB1 translocation occurs in the cholangiocytes of patients with ACLF. Few cholangioles displayed positive HMGB1 staining in the CHB liver tissues (Fig. 1B). Negative HMGB1 or focal positive staining was observed in the normal liver donors (Fig. 1C). In addition, positive HMGB1 staining was also observed in the monocytes, lymphocytes, and hepatocytes of the inflammatory portal areas in the livers of patients with ACLF and CHB but not in the livers of normal donors.

**Figure 2:**
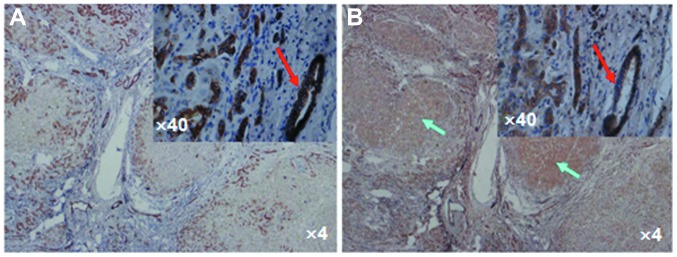
Immunohistochemical staining of HMGB1 and CK7 in cholangiocytes in acute-to-chronic liver failure (ACLF) liver specimens. Newly formed cholangioles were positive for (A) CK7 and (B) HMGB1 in ACLF liver specimens. Red arrow: Positive staining in cholangiocytes. Green arrow: Positive staining of HMGB1 in hepatocytes. Original magnification, ×4 and ×40 (inset). HMGB1, high mobility group box 1.

CK7 was used as a biomarker for cholangiocytes to confirm the cell origin. Positive CK7 staining was observed in the cytoplasm of the cholangiocytes in the newly formed cholangioles. The cholangiocytes of the newly formed cholangioles that were positive for CK7 staining were also positive for HMGB1 staining in the liver samples of ACLF patients (Fig. 2), indicating that the release of HMGB1 from cholangiocytes plays a crucial role in the high serum HMGB1 level in patients with ACLF. The proliferation of large amounts of newly formed cholangioles accompanied by the release of the proinflammatory mediator HMGB1 suggests the possibility of intrahepatic cholestasis caused by inflammation around the cholangioles and bile duct. In addition, positive HMGB1 staining was also observed in the hepatocytes of certain pseudolobules (Fig. 2B), consistent with a previous study (23).

Of the 13 patients with ACLF, 11 showed >67% positive (+++) staining for HMGB1 in the cholangiocytes, and two showed 33–67% positive staining (++) for HMGB1. However, none of the CHB patients showed >33% positive HMGB1 staining (+++~++) in cholangioles; 2 of the 20 CHB patients showed weakly positive (<5%) HMGB1 (+) staining, and 18 of the 20 patients showed negative HMGB1 staining. Almost all the normal donors exhibited negative HMGB1 staining, except for one or two focal positive cholangioles. Thus, HMGB1 release was significantly higher in the patients with ACLF than in the patients with CHB (P<0.001; Table II).

### Active and passive secretion of HMGB1 from TFK-1 cells stimulated with LPS and TNF-α

**Figure 3:**
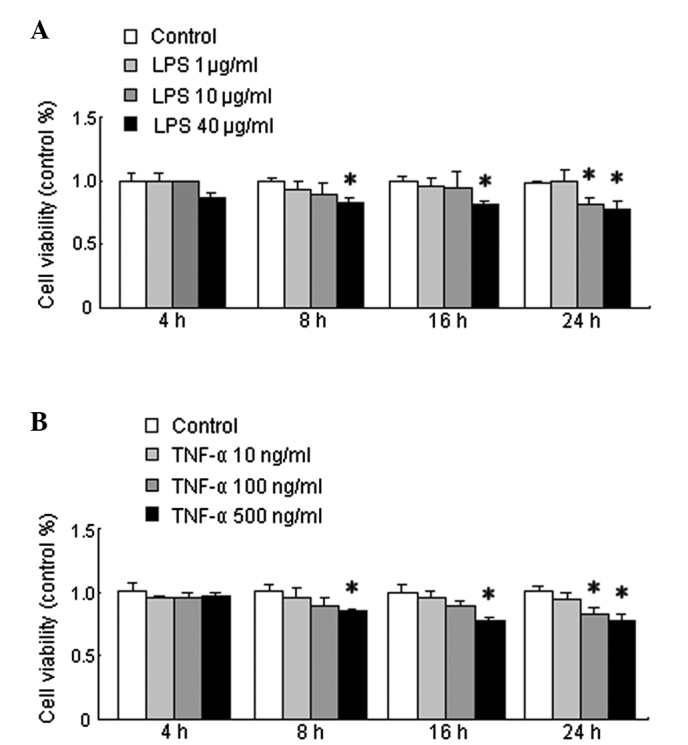
Viability of TFK-1 cells stimulated with LPS or TNF-α. (A) Viability of TFK-1 cells stimulated with various concentrations of LPS at each time point. (B) Viability of TFK-1 cells stimulated with various concentrations of TNF-α at each time point. ^*^P<0.05 compared with the control group. LPS, lipopolysaccharide; TNF, tumor necrosis factor.

**Figure 4:**
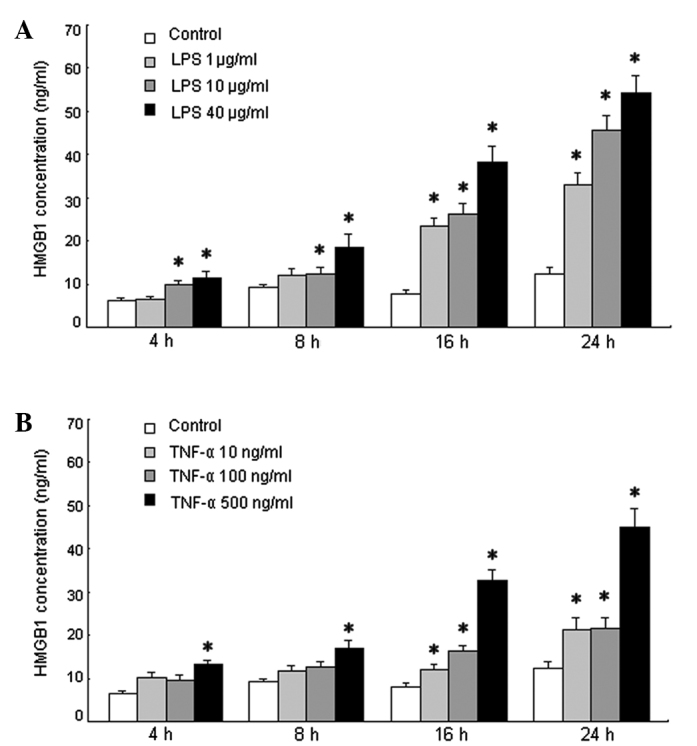
HMGB1 concentration in the culture medium of TFK-1 cells treated with various concentrations of LPS or TNF-α at each time point. (A) HMGB1 concentration gradually increased following stimulation by LPS at each time point compared with that for untreated cells. (B) HMGB1 concentration gradually increased following stimulation by TNF-α compared with that for untreated cells at each time point. ^*^P<0.05 compared with the control group. LPS, lipopolysaccharide; TNF, tumor necrosis factor; HMGB1, high mobility group box 1.

To examine the active secretion of HMGB1 by TFK-1 cells, it was investigated whether LPS and TNF-*α* induced HMGB1 release from TFK-1 cells. Cultured TFK-1 cells were subjected to increasing concentrations of LPS or TNF-α for 4, 8, 16 and 24 h, as described in Materials and methods. The HMGB1 concentration in the medium of the cultured cells was determined at different time points using an ELISA assay simultaneously with an examination of cell viability. As shown in Fig. 3A, there was no difference between the cell viability of the untreated cells and that of the cells treated with LPS for 4 h at each concentration. The viability of the cells treated with LPS for 8, 16, and 24 h at a concentration of 40 μg/ml and for 24 h at 10 μg/ml was significantly decreased compared with that of the untreated cells (P<0.05). The HMGB1 concentration in the medium of the LPS-treated TFK-1 cells at each LPS concentration at each time point is shown in Fig. 4A. The HMGB1 concentration increased after stimulation with LPS at each time point compared with that for the untreated cells (P<0.05).

There was no difference between the cell viability of the untreated cells and the cells treated with TNF-α for 4 h at each concentration. As shown in Fig. 3B, cell viability was significantly decreased in the cells treated with TNF-α for 8, 16 and 24 h at a concentration of 500 ng/ml and for 24 h at 100 ng/ml compared with that of the untreated cells (P<0.05). The HMGB1 concentration in the culture medium of the TNF-α-treated TFK-1 cells at each TNF-α concentration at each time point is shown in Fig. 4B. The HMGB1 concentration gradually increased after stimulation with TNF-α compared with that for the untreated cells at each time point. The HMGB1 concentration in the medium of the cells treated with 500 ng/ml TNF-α was increased significantly at each time point compared with that for the untreated cells (P<0.05). In addition, the HMGB1 concentration in the medium of the cells treated with 10 or 100 ng/ml TNF-α for 16 and 24 h was also significantly increased compared with that for the untreated cells (P<0.05). Therefore, exogenous (LPS) and endogenous (TNF-α) inflammatory stimuli induced HMGB1 release in TFK-1 cell cultures, starting at 4 h post-LPS or -TNF-α stimulation. The release of HMGB1 was not completely dependent on cell death since the cell viability was not significantly altered by 1 or 10 μg/ml LPS or by 10 or 100 ng/ml TNF-α, even at 16 h post treatment (Fig. 3). Furthermore, LPS- or TNF-α-stimulated TFK-1 cells released HMGB1 in a concentration-dependent manner (Fig. 4), starting at concentrations as low as 10 μg/ml LPS (Fig. 4A) or 500 ng/ml TNF-α (Fig. 4B). The increase in HMGB1 concentration was mainly due to active release of HMGB1 by the TFK-1 cells. At slightly cytotoxic dosages, LPS (40 μg/ml) or TNF-α (500 ng/ml) may also cause passive HMGB1 leakage from necrotic TFK-1 cells.

**Figure 5:**
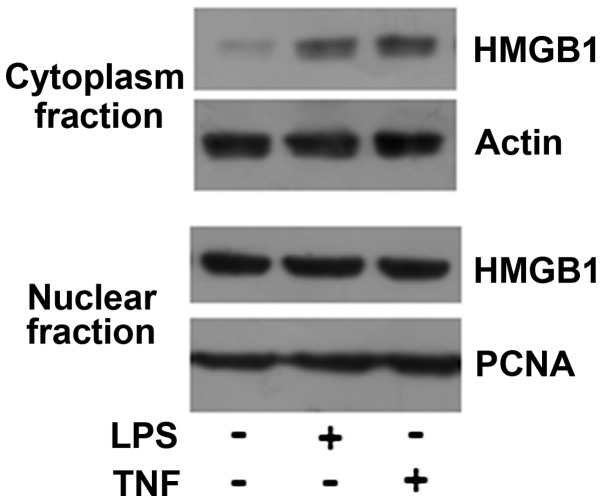
HMGB1 western blot analysis. Following cell fractionation, HMGB1 content in the cytoplasmic or nuclear fraction was determined by western blot analysis. The HMGB1 level in the cytoplasmic fraction increased following stimulation with LPS or TNF-α. HMGB1, high mobility group box 1; PCNA, proliferating cell nuclear antigen; LPS, lipopolysaccharide; TNF, tumor necrosis factor.

Immunohistochemical analysis of the liver tissues of the ACLF patients indicated cytoplasmic translocation of HMGB1 in the cholangiocytes. TFK-1 cells were stimulated with 10 μg/ml LPS or 100 ng/ml TNF-α for 8 h, and the cells were then collected and prepared for cytoplasmic and nuclear fractionation. The results of the western blot analysis are shown in Fig. 5; the HMGB1 level in the cytoplasmic fraction increased following stimulation with LPS or TNF-α. Taken together, these results suggest that endogenous and exogenous inflammatory stimuli can induce HMGB1 nuclear-cytoplasmic translocation in cholangiocytes and active HMGB1 release.

## Discussion

The most common clinical manifestation of liver failure in Asia, particularly in China, is ACLF, which is defined as an acute episode of liver function decompensation in a liver that is already impaired by chronic hepatitis B (24). Although the etiology of ACLF varies between Western and Eastern countries, the clinical manifestations are remarkably similar, suggesting that the various pathogenic factors induce common patterns of innate immune responses (25). In addition, many other proinflammatory cytokines, including TNF-α and IL-6, may play a common role in the pathophysiology of ACLF (26). It has been reported that monocytes/macrophages actively release HMGB1 in response to exogenous stimuli, such as LPS, or endogenous inflammatory stimuli, including TNF-α and IL-1β (11). Active HMGB1 release has been demonstrated in non-immune cells including pituicytes and enterocytes (27,28). HMGB1 release has been observed in hepatocytes from patients suffering from various liver diseases (23,29), and the cytoplasmic translocation of HMGB1 has been observed in patients with acute liver failure (23). Inflammation of the cholangioles is also important in ACLF; therefore, whether HMGB1 is released from cholangiocytes in patients suffering from ACLF was investigated.

In this study, cytoplasmic translocation of HMGB1 was observed in cholangiocytes from patients with ACLF caused by HBV infection. Furthermore, the experiments revealed that active nuclear-cytoplasmic HMGB1 translocation and release occurred in TFK-1 cells upon stimulation with exogenous (LPS) and endogenous (TNF-α) inflammatory stimuli.

In China, a large proportion of ACLF cases are caused by HBV infection (1); thus, the present study evaluated the cytoplasmic translocation of HMGB1 in patients with ACLF caused by hepatitis B. Consistent with previous reports, histopathological changes were observed, including massive, sub-massive, or bridging necrosis with immune cell infiltration in the cholangiocytes. Notably, HMGB1 was clearly observed in the cholangiocytes, particularly in newly formed cholangioles, in samples from patients with ACLF caused by HBV infection but not in samples from patients with chronic HBV infection or normal donors. One limitation of this clinical study is that the liver tissue samples were obtained during liver transplantation surgery and thus did not represent the early clinical manifestation immediately following the onset of ACLF.

HepG2 cells (human hepatocellular carcinoma cell line) can be induced to release HMGB1 by treatment with LPS or TNF-α in a time- and dose-dependent fashion (23). HMGB1 may also be released passively following necrosis (10,30) and apoptosis, depending on the cell type (31,32). It has also been reported that human gingival fibroblasts release HMGB1 protein through both active and passive pathways (32). In the present study, positive HMGB1 staining was predominantly observed in the cytoplasm and extracellular areas of cholangiocytes in the most inflamed and portal area in the samples from the patients with ACLF, indicating cytoplasmic translocation of HMGB1 in these cells. Furthermore, HMGB1 translocation from the nucleus to the cytoplasm was confirmed by western blot analysis in TFK-1 cells incubated with LPS and TNF-α. It has been widely suggested that re-localization and accumulation of HMGB1 in the cytoplasm is a necessary step for its extracellular release, raising the possibility that activated cholangiocytes, particularly newly formed ones, could be a source of extracellular HMGB1, which might contribute to the inflammatory response during ACLF. During liver injury/failure, HMGB1 may be released as a danger signal from the activated or damaged cholangiocytes as well as from immune cells and necrotic cells.

Clinical signs and symptoms may not indicate whether the stimulating event was active or passive because complications, particularly infections, usually accompany ACLF. Of the 13 ACLF patients investigated, all were positive for serum HBsAg, and 11 were positive for HBsAg in liver tissue. In addition, five patients suffered from bacterial infections, four from ascites, and one from a gastrointestinal tract hemorrhage. Thus, HMGB1 may be synergistically released by the above two pathways as a result of infection and cell damage, leading to exacerbated inflammation and injury of the bile duct, particularly the newly formed cholangioles, intrahepatic cholestasis, and hyperbilirubinemia during the process of ACLF. HMGB1 release was observed in the cholangiocytes, particularly in the newly formed cholangioles, in the liver tissues of ACLF patients. Active and passive release of HMGB1 was observed in TFK-1 cells induced with LPS or TNF-α *in vitro*. Therefore, HMGB1 may act as a late inflammatory mediator triggered by LPS and TNF-α in the inflammatory process of ACLF. Cellular and ductular cholestasis indicate acute injury of the liver. Features of cholestasis and bile duct proliferation are very common in patients with acute liver injury, particularly in patients with ACLF. The release of HMGB1 from the newly formed cholangiocytes was confirmed by CK7 staining in the inflamed portal area. It may be hypothesized that the increased inflammation triggered by HMGB1 and/or TNF-α could be at least partly responsible for intrahepatic cholestasis and could further lead to hyperbilirubinemia in patients with ACLF.

To the best of our knowledge, this is the first study to demonstrate that HMGB1 is actively released from cholangiocytes, particular from newly formed cholangioles, in ACLF. As an effective inflammatory mediator of TNF-α or endotoxin (LPS), HMGB1 may progressively exacerbate the inflammation and damage of the bile duct, intrahepatic cholestasis, and hyperbilirubinemia during the early stages of ACLF. Infection is the most common cause of exacerbated hyperbilirubinemia in the clinic. The prevalence of ductular bilirubinostasis in biopsy specimens from patients with developed infections is significantly higher than in those without developed infections (33). Since HMGB1 is elevated during infection, strategies to reduce HMGB1 activity using antibodies may be therapeutically effective (34,35). Duan *et al* (36) indicated that an enhanced serum level of HMGB1 was associated with the development of HBV-related ACLF in patients with CHB. The strong correlation between HMGB1 and AST levels suggest that HMGB1 may be a useful prognostic marker for the development of ACLF.

The present results demonstrate the nuclear-cytoplasmic translocation of HMGB1 in the cholangiocytes of patients with ACLF caused by HBV infection as well as in TFK-1 cells upon stimulation with either exogenous (LPS) or endogenous (TNF-α) inflammatory stimuli. These observations raise important questions regarding the potential pathogenic roles of HMGB1 in cholangiocytes in ACLF and suggest that HMGB1 may be a therapeutic target.

## Figures and Tables

**Table I:** Baseline characteristics of the patients in the ACLF and chronic hepatitis B groups.

Characteristics	ACLF (n=13)	Chronic hepatitis B (n=20)	P-value
Age (years)	41.84±12.31	28.20±7.01	<0.005
Gender (female/male)	3/10	8/12	0.456
ALT (U/L)	165.75±221.51	162.91±135.73	0.968
AST (U/L)	158.28±158.75	91.42±71.59	0.157
TB (μmol/l)	428.76±214.40	19.49±13.17	<0.005
DB (μmol/l)	232.56±115.99	6.89±6.65	<0.005
ALB (g/l)	34.16±4.20	38.60±5.22	0.025
PT (sec)	28.73±6.74	12.51±0.70	<0.005
PTA (%)	26.47±11.52	95.06±11.48	<0.005

ACLF, acute-to-chronic liver failure; ALT, alanine aminotransferase; AST, aspartate aminotransferase; TB, total bilirubin; DB, direct bilirubin; ALB, albumin; PT, prothrombin time; PTA, prothrombin activity.

**Table II:** HMGB1 expression in cholangiocytes in the liver tissues of patients with ACLF and CHB.

	HMGB1 expression			
	
Patient groups	−	+	++	+++
ACLF (n=13)	0	0	2	11
CHB (n=20)^a^	18	2	0	0
P-value			<0.001	

a Calculated from non-parameter test.

ACLF, acute-on-chronic liver failure; CHB, chronic hepatitis B; HMGB1, high mobility group box 1.
